# Economic evaluation of the Hepatitis C virus elimination program in the country of Georgia, 2015 to 2017

**DOI:** 10.1111/liv.15431

**Published:** 2022-10-04

**Authors:** Irina Tskhomelidze, Shaun Shadaker, Tinatin Kuchuloria, Lia Gvinjilia, Maia Butsashvili, Muazzam Nasrullah, Tamar Gabunia, Amiran Gamkrelidze, Vladimer Getia, Lali Sharvadze, Tengiz Tsertsvadze, Jaba Zarqua, Shota Tsanava, Senad Handanagic, Paige A. Armstrong, Francisco Averhoff, Peter Vickerman, Josephine G. Walker

**Affiliations:** 1 Task Force for Global Health, Tbilisi, Georgia; 2 Ivane Javakhishvili Tbilisi State University, Tbilisi, Georgia; 3 Division of Viral Hepatitis, Centers for Disease Control and Prevention, Atlanta, USA; 4 Neolab, Tbilisi, Georgia; 5 Ministry of Internally Displaced Persons from the Occupied Territories, Labour Health and Social Affairs of Georgia, Tbilisi, Georgia; 6 National Center for Disease Control and Public Health, Tbilisi, Georgia; 7 Hepa Clinic, Tbilisi, Georgia; 8 Infectious Diseases, AIDS and Clinical Immunology Research Center, Tbilisi, Georgia; 9 Mrcheveli Clinic, Tbilisi, Georgia; 10 Population Health Sciences, University of Bristol, Bristol, UK

## Abstract

**Background and Aims:**

In 2015, the country of Georgia launched an elimination program aiming to reduce the prevalence of Hepatitis C virus (HCV) infection by 90% from 5.4% prevalence (~150 000 people). During the first 2.5 years of the program, 770 832 people were screened, 48 575 were diagnosed with active HCV infection, and 41 483 patients were treated with direct-acting antiviral (DAA)-based regimens, with a >95% cure rate.

**Methods:**

We modelled the incremental cost-effectiveness ratio (ICER) of HCV screening, diagnosis and treatment between April 2015 and November 2017 compared to no treatment, in terms of cost per quality-adjusted life year (QALY) gained in 2017 US dollars, with a 3% discount rate over 25 years. We compared the ICER to willingness-to-pay (WTP) thresholds of US$4357 (GDP) and US$871 (opportunity cost) per QALY gained.

**Results:**

The average cost of screening, HCV viremia testing, and treatment per patient treated was $386 to the provider, $225 to the patient and $1042 for generic DAAs. At 3% discount, 0.57 QALYs were gained per patient treated. The ICER from the perspective of the provider including generic DAAs was $2285 per QALY gained, which is cost-effective at the $4357 WTP threshold, while if patient costs are included, it is just above the threshold at $4398/QALY. All other scenarios examined in sensitivity analyses remain cost-effective except for assuming a shorter time horizon to the end of 2025 or including the list price DAA cost. Reducing or excluding DAA costs reduced the ICER below the opportunity-cost WTP threshold.

**Conclusions:**

The Georgian HCV elimination program provides valuable evidence that national programs for scaling up HCV screening and treatment for achieving HCV elimination can be cost-effective.

## Lay summary

Scaling up testing and treatment for Hepatitis C virus (HCV) is cost-effective on a national scale in the country of Georgia, but cost-effectiveness is strongly affected by drug costs. This provides valuable guidance for the next phases of the Georgia HCV elimination program as well as helps other countries to decide how to implement national HCV treatment programs.

## 1 ∣ INTRODUCTION

Hepatitis C virus (HCV) is a global public health problem: in 2015, 71 million people were living with HCV infection, and 1.75 million people were infected annually.^1^ People with HCV are at high risk for developing chronic liver disease, cirrhosis and hepatocellular carcinoma (HCC).^2^ To tackle this problem, in May 2016, the World Health Assembly signed the Global Health Sector Strategy (GHSS) on Viral Hepatitis with the goal of eliminating viral hepatitis as a public health threat by 2030.^3^

With curative direct-acting antiviral (DAA) treatments, it is possible to achieve a drastic reduction in HCV prevalence and incidence through treatment alongside prevention measures.^4^ From 2015 to 2019, 9.4 million (7.5 million–11.7 million) people were treated for hepatitis C using DAAs.^5^ Owing to international licensing laws, the variation in cost for drugs across countries is not directly associated with the country's gross domestic product (GDP) or capacity to pay, therefore, DAA prices are inaccessible in many countries,^6^ presenting a large barrier for access to hepatitis C treatment.^7^ In 2015, prices were highest in central and Eastern European countries (1.09–1.63 times USA cost) when adjusted for purchasing power parity.^8^ In addition to drug costs, scaling up treatment is likely to require a large investment in medical infrastructure and case-finding^9^ which may be difficult in the absence of large financial donor programs or without novel funding models.^10^

In the country of Georgia, a national HCV seroprevalence survey (NSS) in 2015 estimated that 7.7% of the adult population were HCV antibody (anti-HCV) positive and HCV RNA prevalence was 5.4% (approximately 150 000 people).^11^ Georgia was the first country to launch a national HCV elimination program, setting an ambitious elimination target of reducing the prevalence of chronic HCV infection by 90% by 2020 through scaling up treatment and prevention interventions.^12^ Political commitment, investment, and community network empowerment, alongside collaboration with partners such as Gilead Sciences (who donated DAA drugs), were important components of developing the comprehensive national program.^13-15^ Although Georgia did not reach their elimination target by 2020,^16^ they are one of only 12 countries estimated to be on track to eliminate viral hepatitis by 2030 under the GHSS targets, which would reduce HCV incidence by 80%, and the number of attributable deaths by 65%.^17^

From the launch of the elimination program in April 2015 until 30 November 2017, 41 483 people initiated treatment for active HCV infection. Initially, patients known to have severe liver disease were prioritized for treatment,^12^ but after June 2016, treatment became available for all HCV-infected citizens and permanent residents in Georgia. Since November 2017, additional changes were made in diagnostic and treatment pathways: HCV core antigen (HCVcAg) testing was introduced in December 2017^18^ followed by the removal of patient co-payment for diagnostics before treatment and during treatment monitoring in 2019.

Our objective was to model the cost-effectiveness of the screening and treatment undertaken as part of the HCV elimination program in Georgia from the start of the program in April 2015 through to November 2017, which captures treatment of patients who already knew they were infected, alongside the scale-up of community and health service-based screening.^19^ This will be relevant to other countries aiming to introduce or scale-up widespread treatment access towards HCV elimination where access has previously been limited.

## 2 ∣ MATERIALS AND METHODS

We conducted a model-based cost-effectiveness evaluation (cost-utility analysis) of the HCV elimination program in Georgia. We calculated the incremental cost-effectiveness ratio (ICER) of HCV screening, diagnosis and treatment initiated between April 2015 and November 2017 compared to no treatment, in terms of cost per quality-adjusted life year (QALY) gained. We assume no treatment as the counterfactual because prior to the elimination program DAAs were only accessible through the private market and very limited. Costs are reported in 2017 US dollars, and all costs and outcomes were measured over 2015–2039 (25 years), with a 3% discount rate used for costs and outcomes as in standard practice.^20^ We evaluated the cost from the perspective of the Ministry of Internally Displaced Persons from the Occupied Territories, Labour, Health and Social Affairs of Georgia (MOH, the primary payer), including the costs to private clinics and other healthcare providers. Costs to the patient (co-payment was required) were accounted for separately, as were the cost of donated DAA drugs. To evaluate the cost-effectiveness of the program, the ICER was compared to potential willingness-to-pay thresholds of $4357 per QALY gained (standard threshold of 1x GDP per capita in 2017 USD^21^) and $871 per QALY gained (estimated opportunity cost-based threshold of 20% GDP per capita^22^).

### 2.1 ∣ Overview of the HCV elimination program

The screening component of the HCV elimination program included antibody testing in a variety of settings including designated screening sites, primary healthcare facilities, blood banks, harm reduction sites and inpatient facilities. Following a positive HCV antibody test (anti-HCV), patients were tested for viremia (RNA; nucleic acid testing) to diagnose an active infection. Testing for viremia was conducted at one of 15 laboratories (as of December 2017)^23^ followed by baseline pre-treatment evaluation for patients diagnosed with active infection. Infected patients were evaluated for liver damage using the FIB-4 score calculated from age, AST/ALT and platelets; those with values <1.45 were assumed to not have cirrhosis, while those with results >3.25 were assumed to have cirrhosis. Individuals with a FIB-4 score between 1.45 and 3.25 were evaluated for cirrhosis by elastography. Following pre-treatment evaluation, patients were initiated on treatment with a DAA-based regimen (with or without ribavirin or interferon) lasting 12, 20, 24, or 48 weeks depending on clinical condition,^13^ and received regular monitoring during the treatment period. Sustained virological response (SVR) was assessed by viral load test between 12 and 24 weeks after treatment completion.

Some antibody screening and viremia testing was available prior to the launch of the elimination program, which is defined as beginning on 28 April 2015, when the first treatments were given. We exclude the cost of screening and diagnostic tests prior to the elimination program owing to a lack of data, but this only accounted for 4% of the 48 575 patients diagnosed with viremic infection included in this analysis. Patients were treated at either one of 27 designated treatment centres established under the elimination program, a specialized infectious disease hospital (including those diagnosed with HIV co-infection), or in prison.^23^ Adults aged ≥18 years were eligible for treatment, with a small number of children treated on a case-by-case basis by the elimination program's clinical committee.

### 2.2 ∣ Model description

We used a previously described dynamic compartmental model of HCV transmission, disease progression and treatment in Georgia to project the cost and impact of treatment of 41 483 patients during the study period.^24^ The model stratifies the population by HCV infection and treatment states; liver disease progression [none/mild, moderate liver disease, compensated cirrhosis, decompensated cirrhosis (DC) or hepatocellular carcinoma (HCC)]; age (nine categories from birth to 50+ years); sex and injecting drug use status [people who inject drugs (PWID) currently, previously injected, or never injected drugs]. HCV-related death occurs in the DC and HCC stages, and PWID also have a lower life expectancy than people who previously or never injected drugs. The model accounts for different risks of HCV transmission or re-infection for PWID compared to the general population, including how risk has changed over time owing to harm reduction programs and other preventive measures. The dynamic nature of the model allows for capturing individual-level benefits of reduced disease progression as well as population-level benefits of new infections averted by HCV treatment. The number of individuals treated per month according to liver disease stage was incorporated into the model, assuming that treatments are distributed equally to PWID and the general population, and by age and sex.

The model was calibrated using Approximate Bayesian Computation to Georgian data on HCV prevalence by age and sex from the 2015 National Serosurvey (NSS) as well as demographics and prevalence of HCV infection among PWID over time from a series of Georgian bio-behavioural surveys,^25^ with prior distributions for other parameters from international literature. The model calibration allows for uncertainty in model parameters propagated into model projections and produced 554 baseline model fits. For each of these model fits, we applied cost and utility estimates (described below) to patients according to their treatment and liver disease stage for each year (2015–2039). The model fits were then run for the intervention and counterfactual (no treatment) scenarios in order to estimate the impact of treatment on morbidity and mortality in the population. Results are presented based on the mean value and 95% credible interval (CrI, 2.5 and 97.5 percentiles across the model output) of the 554 model fits.

### 2.3 ∣ Costing methods

We conducted a top-down costing analysis to estimate the average cost of screening, diagnosis and treatment per patient as well as the annual cost of care for patients with liver disease. Unit costs were gathered from national government reimbursement schemes for healthcare providers and elimination program records, accounting for provider costs (MOH and health facility) and patient co-payments. The cost of screening, diagnosis and treatment for HIV-HCV co-infected individuals or those in prison was funded through different state programs, however, we assume the same cost for those treated within these groups. The average cost of screening, diagnosis and treatment for the provider and the patient was calculated by accounting for changes in MOH reimbursement policy, treatment regimen and co-payments during the study period, during which time the proportion of treatment monitoring costs paid by the national program increased and patient out-of-pocket costs decreased. All the costs were recorded in Georgian Lari (GEL), converted into USD using yearly exchange rates for 2015–2017 and then inflated to 2017 USD, the latest year for which costs were recorded.^26^ We do not sample uncertainty in unit costs or resource use as we do not have detail on patient-level variation in costs, however, we vary cost inputs in deterministic sensitivity analysis to account for the proportion of patients in different groups, as described below.

#### 2.3.1 ∣ Resource use

The number of patients screened, diagnosed and treated was extracted from program data in the national elimination program's screening registry and the treatment database (Elimination-C) by calendar year (2015, 2016 or 2017) within the study period. The proportion of patients requiring elastography testing and receiving each drug regimen each year was extracted from the treatment database and MOH reimbursement records and used to weight the average cost per patient treated each year. In addition, patients classified as socially vulnerable (defined as a person who receives assistance from the state to create basic living conditions, whose income rating equals or is lower than the threshold established by the Government of Georgia) were eligible for reduced co-payments, and the average costs to the MOH and the patient were weighted according to the proportion of socially vulnerable patients treated.

#### 2.3.2 ∣ Unit costs

Screening and treatment-associated costs within the elimination program were divided by payers, the MOH and the healthcare provider, versus patients. Treatment costs gathered from the MOH reimbursement records include ribavirin and interferon (non-DAA drug costs) where applicable, costs of tests and procedures related to baseline assessment for treatment initiation, on-treatment monitoring and SVR testing but excluded DAA drug costs as these were donated (Data S1). MOH reimbursement costs to healthcare providers were assumed to cover the full cost, as, since June 2016, these have accounted for overheads, such as equipment upgrades for treatment provider clinics and administrative costs. In the base case, economic costs of the DAA drugs were based on generic drugs sourced from India (Table 3), and market prices were gathered from interviews with healthcare providers treating foreign country citizens outside of the HCV elimination program.

As the model does not directly account for screening and diagnostic testing, we estimated the cost of diagnosing a patient with active HCV per patient treated according to the number of patients screened and the anti-HCV and viremic positivity rates during each year of the study period, extracted from the Elimination-C database.

Other indirect (fixed) costs included outreach and program promotion, drug security logistics and other overheads which were provided by MOH and NCDC records. However, we were not able to estimate fixed costs related to management for adverse events, treatment database development and maintenance, local and international technical expert salaries and annual technical advisory group meetings and planning workshops, where international and national experts came together to evaluate and guide next steps for the elimination program.

#### 2.3.3 ∣ Cost of liver disease care

Annual healthcare costs attributable to liver disease progression were accounted for in the model in order to capture long-term healthcare costs averted by the elimination program. Data on annual inpatient costs for patients with advanced liver disease caused by active HCV infection were gathered from the database held by the state program on the management of infectious diseases (Data S1 and Table S2). The state program covered treatment for chronic viral hepatitis, categorized as either with highly active pathological process; with cirrhosis; or with cirrhosis, ascites and/or encephalopathy and/or hepato-renal syndrome; with different proportional co-payments applied for different groups. The number of patients treated was compared to the number of patients modelled in each liver disease stage to estimate the percentage of patients at each fibrosis/cirrhosis stage that access liver disease care. As more recent data on liver disease care were not available, we assume the proportion of patients with liver disease accessing care annually remained stable from 2017 onwards.

### 2.4 ∣ Health-related quality of life

Quality-adjusted life year (QALY) weights for liver disease stages were estimated using EQ-5D-5L survey (EuroQol) questionnaires.^27^ Data were collected from a subset of 274 HCV-infected patients in Tbilisi that were treated within the elimination program and enrolled in a study evaluating long-term outcomes of individuals with liver disease, with the EQ-5D-5L survey completed by each patient prior to treatment initiation. EQ-5D-5L results for patients with mild or moderate liver disease, or compensated cirrhosis were converted to QALY weights based on the EQ-5D-5L Index Value Calculator which uses EQ-5D-3L crosswalk values from the United Kingdom,^27,28^ Liver disease state was calculated based on transient elastography or FIB-4 score, with METAVIR^29^ F0-F1 classed as mild, F2-F3 as moderate and F4 as cirrhosis. Patients with cirrhosis had lower average health state values (0.74, 95% confidence interval 0.68–0.80) compared to patients with moderate (0.81, 0.77–0.85) or mild (0.83, 0.81–0.85) liver disease. The mean weight value for each group was used in the model, as shown in Table S5.

No local results were available for DC, HCC or post-SVR stages for which we used QALY estimates from a previous study in the Canadian population.^30^ In the base case, we assumed that PWID experiences the same quality of life weights as the rest of the population. We assume that patients with mild or moderate liver disease or compensated cirrhosis experience an improvement in health-related quality of life after SVR, while those with DC or HCC do not, as post-SVR data were drawn from patients with a median METAVIR score of F2.^30^

### 2.5 ∣ Sensitivity analysis

In order to address the uncertainty around key parameters and generalize our findings, we conducted several sensitivity analyses by varying individual parameters from the base case. We evaluated the change in the ICER from different perspectives including or excluding costs paid out of pocket by the patient as well as by the MOH, and to add the full (list) price, use a minimal production-based price^31^ (Data S1), or to exclude the price of DAA drugs. We changed the QALY weights for mild, moderate and compensated cirrhosis based on the same external study from which advanced liver disease weights were drawn,^30^ instead of those measured in Georgia. The values for mild and moderate liver disease in Georgia were higher than the alternative QALY weights from the literature (0.76 for mild and moderate liver disease), but the value for cirrhosis was the same.^30^ We also scaled baseline QALY weights to account for a lower quality of life in PWID, with QALY weights for each disease category multiplied by 0.79 as in a previous study.^4^ We also calculated the cost to the MOH if all or none of the patients were classed as socially vulnerable (15.3% in 2015, 9.3% in 2016 and 12.3% in 2017 in the base case). Finally, we varied the discount rate to 0% and 7% (3% in the base case) and reduced the time horizon to 2015–2025 (2015–2039 in the base case).

In addition, we assessed the variation in total costs and outcomes across the 554 model fits, in which parameters for disease progression and transmission vary, in order to calculate the percent of model runs with ICERs below each WTP threshold.

## 3 ∣ RESULTS

### 3.1 ∣ Impact of the elimination program

**FIGURE 1:**
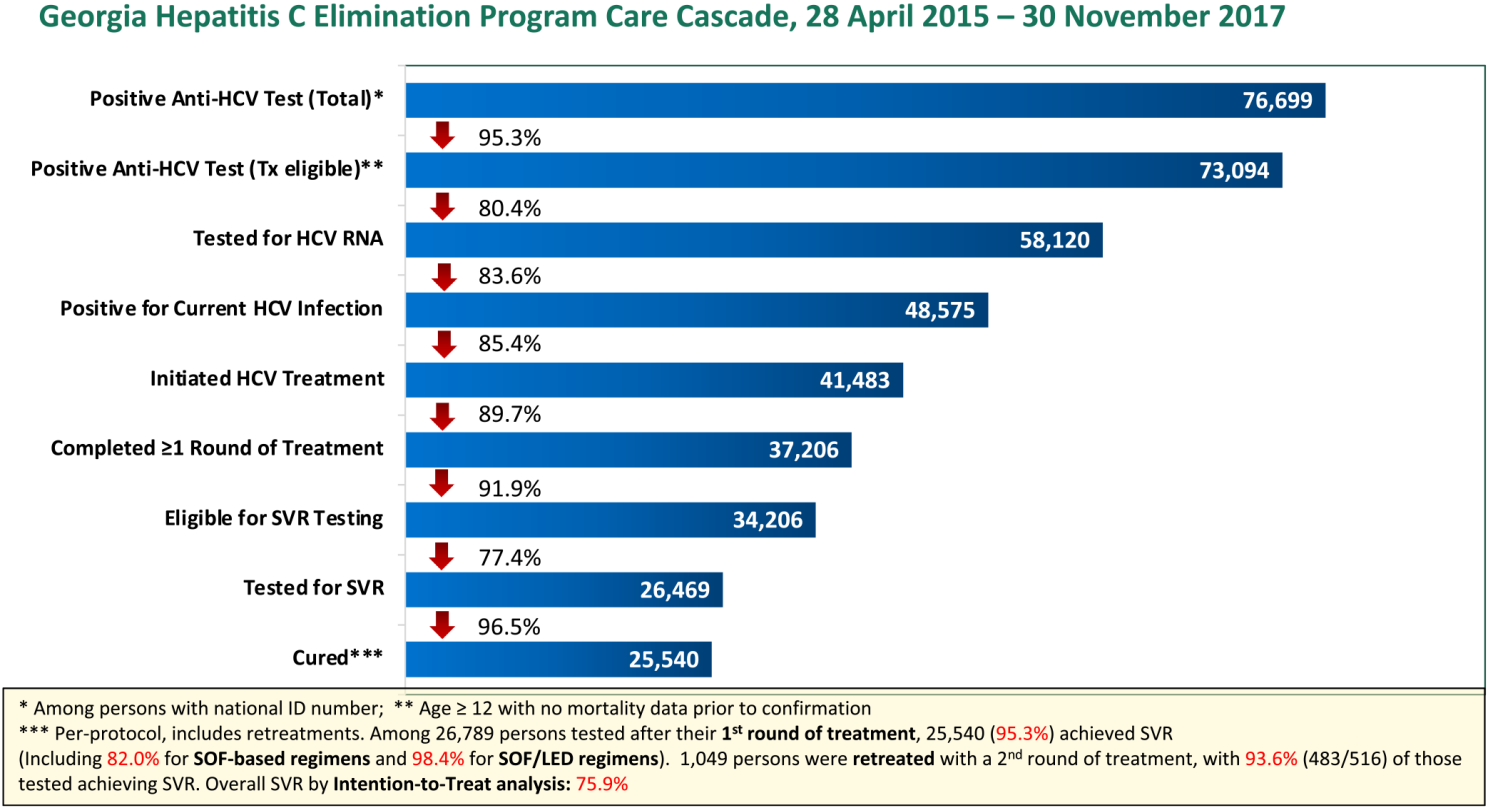
Care cascade of patients receiving positive anti-HCV test during the study period.

During the study period from April 28, 2015, to November 30, 2017, 770 832 unique patients were screened, and 41 483 patients were treated (Table 1 and Figure 1). All treated patients received either sofosbuvir or ledipasvir/sofosbuvir-based regimens (Sovaldi or Harvoni, Gilead Sciences, Foster City, CA, USA), in some cases in combined therapy with PEG Interferon (9%) and/or Ribavirin (55%) (Table 2).

The model projects that the 41 483 treatments given by November 2017 led to a mean of 100 (95% CrI 51–156) deaths averted and 1655 (95% CrI 835–2813) new infections averted by November 2017, which increases to 2662 deaths (95% CrI 1689–3678) and 16 826 (95% CrI 8027–29 290) new infections averted if outcomes are counted over 25 years. In total (without discounting), from 2015 to 2039, 38 031 QALYs (95% CrI 26 382–51 225) were gained by the intervention. In addition, over the same time period, 22 024 (6062–41 389) years lived with compensated cirrhosis, 11 073 (6852–16 130) years lived with decompensated cirrhosis, and 2485 (505–4583) years lived with HCC were prevented.

### 3.2 ∣ Cost of HCV screening and treatment

The total cost of care and treatment is $25 354 509 ($16 015 324 to the MOH and $9 339 185 to patients), plus $43 234 703 for generic DAAs (not discounted). Without including the cost of DAA drugs, the average cost per patient treated for 41 483 treatments was $386 to the MOH and $225 to the patient, covering the cost of screening, diagnosis, pre-treatment evaluation, monitoring, non-DAA drugs and overheads, adding the cost of DAAs is an additional $1042 per patient. The largest contributors to treatment costs after the DAA costs are non-DAA drugs and pre-treatment monitoring. Screening costs per treated patient changed each year in the study period owing to the ratio of patients screened to treated varying, from 8.4 to 7.3, and 40.1 individuals screened per treated patient in 2015, 2016, and 2017 respectively.

### 3.3 ∣ Cost of HCV-related liver disease

**FIGURE 2:**
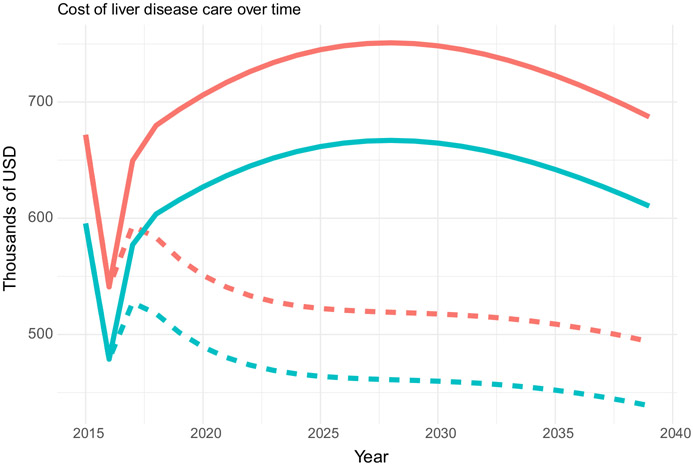
Cost of liver disease care over 25 years (2015–2039) to MOH only (green) or including the cost to patients and to MOH together (red), with (dashed line) and without (solid line) the scale-up in DAA treatment, in non-discounted 2017 USD.

The cost of liver disease care over 25 years decreases owing to the 41 483 treatments given in the study period compared to the no-treatment scenario (Figure 2). Without HCV treatment, the cost over 25 years in non-discounted 2017 USD would be a total of $15 835 974 (CrI $13 722 974–$18 556 888, note that uncertainty bounds are based on uncertainty in projected liver disease, as input costs are not varied) for the MOH and $1 996 661 (CrI $1 730 935–$2 340 329) for patients, while with the scale-up in HCV treatment, this reduces to $11 825 906 (CrI $10 213 016–$13 716 326) to the MOH and $1 491 359 (CrI $1 290 114–$1 729 491) to the patient over the same time period, a saving of 25%.

### 3.4 ∣ Cost-effectiveness

Under the base case with 3% discounting and including the generic cost of DAA drugs, the MOH pays $2285/QALY gained (Table 4). This is cost effective at the standard threshold of 1xGDP per capita or $4357/QALY but above the opportunity-cost threshold of 20% GDP or $871/QALY. Across the model fits in the base case, 99.8% of ICER estimates are below the 1xGDP/QALY threshold but none are below the 20% GDP/QALY threshold.

**FIGURE 3:**
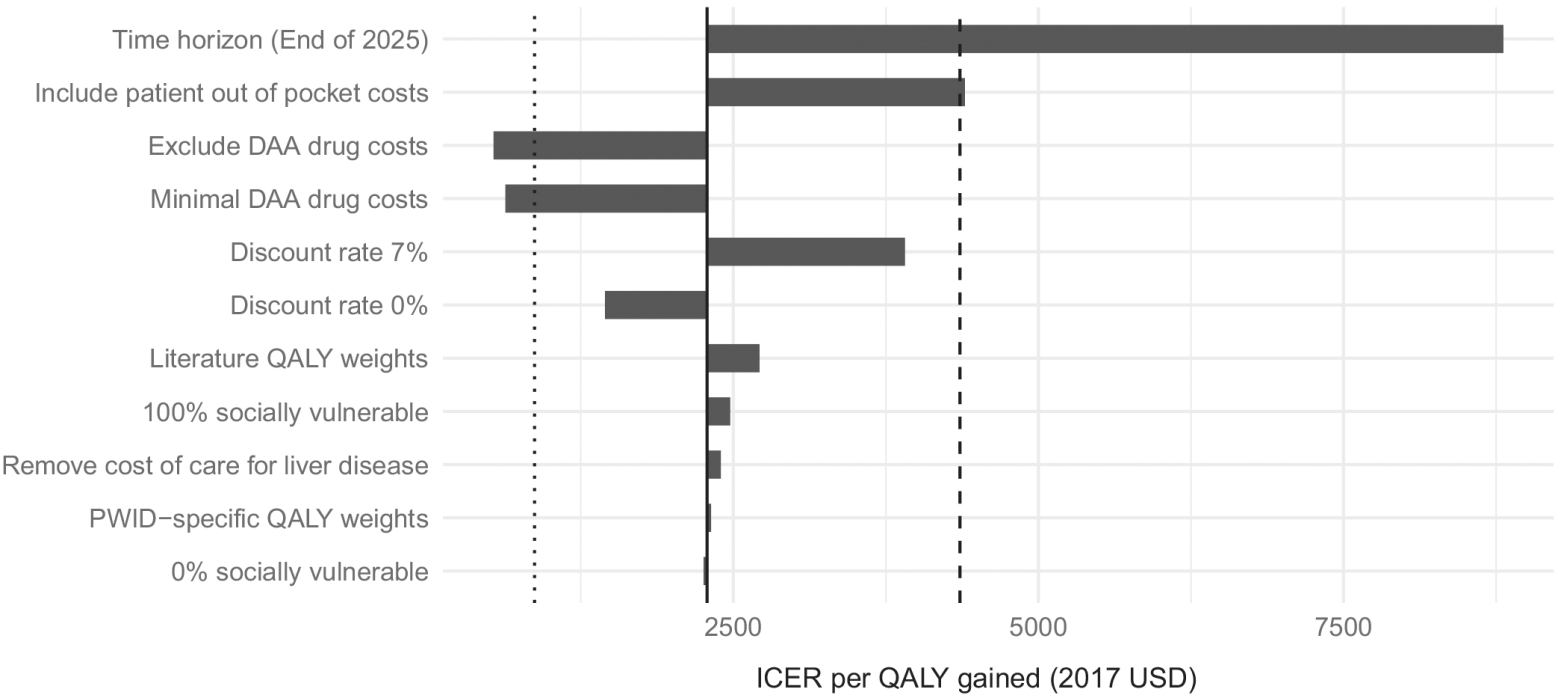
Sensitivity analysis ‘tornado plot’ showing variation in ICER under different scenarios. Full DAA cost scenario not shown (ICER $136 052/QALY). The dashed line shows the WTP threshold of 1x GDP per capita, and the dotted line shows the alternative opportunity cost-based WTP threshold of 20% GDP per capita.

The intervention remains cost-effective at the 1xGDP threshold under most scenarios except when a short-time horizon (to the end of 2025) is used, or if the full list price of DAAs is used (Figure 3). If all patient out of pocket costs are accounted for, the ICER is just above the cost-effectiveness threshold at $4398. Reducing or excluding DAA costs reduced the ICER below the 20% GDP cost-effectiveness threshold.

## 4 ∣ DISCUSSION

Our analysis suggests that the first 2.5 years of the HCV elimination program were cost-effective compared to the standard GDP per capita WTP threshold at $2285/QALY when accounting for generic DAA costs available in Georgia. If the list price were paid for DAAs, then it would not be cost effective. Reducing DAA costs to a minimal production cost level would make the intervention cost-effective at the lower WTP threshold. The generic DAA costs may not have been available in 2015, and even in 2021, many countries do not have access to affordable generic DAAs.^32^ Cost of treatment drugs remains one of the key drivers of cost, especially early in a program and when there are an anticipated high number of people who will need treatment. Developing strategies for reducing cost of treatment (e.g., cost negotiation, obtaining generic medications) will be critical in ensuring a program is feasible for a country. In the Georgia program, the donation of highly effective DAAs has made the program highly cost-effective for the government. In addition, the intervention is not cost-effective when all patient costs are included, which is important to note as more patient costs have been covered by the MOH since 2018 in order to encourage participation in the program. To balance this out and keep the intervention cost effective, other costs could be saved, such as by simplifying the pathway of care and reducing treatment monitoring costs and diagnostic costs; such strategies have also been incorporated in the latter stages of the elimination program and should be evaluated in future research.

### 4.1 ∣ Strengths and limitations

The main strength of this study is that it is the first cost-effectiveness study of a national HCV elimination program. This study provides estimates of costs and health outcomes for a hepatitis C elimination initiative, which is useful information for other governments considering similar strategies. Results for cost-effectiveness were presented including costs of all screening, pre-treatment evaluation, treatment monitoring and post-treatment laboratory tests. We account for the cost covered by the program and the co-financing required from patients, and how this changed over the first 2.5 years of the program. We built on a previously validated dynamic model of HCV transmission^24^ to calculate the morbidity and mortality benefits of providing HCV treatment.

A key limitation of the study is that as the strategy for the HCV elimination program changed over time, we were only able to evaluate the first 2.5 years of the program, during which many patients who already knew their HCV status and had more advanced liver disease accessed treatment. However, a similar situation is likely to exist in other countries setting out to introduce national HCV treatment programs, and so the results are likely to be relevant. We also assume that the proportion of patients accessing care for liver disease will remain stable in the future, though this may increase owing to awareness raising around HCV infection alongside the elimination program. Some costs were not available to include, such as screening that occurred prior to the elimination program launch, database development and management of adverse events.

After this study period, considerable regulatory changes were made to improve access and linkage to free-of-charge HCV viremia testing by introducing core antigen testing and reducing (and then completely removing) costs to patients throughout the treatment pathway.^18^ In addition, some decentralization of HCV care to primary healthcare and harm reduction centres was introduced,^33^ potentially reducing the cost of treatment. Further studies are needed to evaluate what case-finding or linkage to care strategies are most cost-effective as the HCV elimination program is scaled up and reaches more people who were not aware of their infection prior to the program. For example, previous studies have found that DAA treatments are more likely to be cost-effective in PWID when implemented alongside harm reduction programs;^34-36^ harm reduction and other preventative measures have been a priority in Georgia's HCV elimination program and are likely to increase the impact of treating HCV infection in PWID.

### 4.2 ∣ Comparison to other studies

There are studies estimating the cost-effectiveness of HCV screening and treatment interventions using DAAs, though the literature in the context of large-scale elimination programs is lacking.^37^ Additionally, there is little data on the cost-effectiveness of HCV screening and treatment in lower- and middle-income countries.^38,39^ Generally, DAAs are considered to be cost-effective or cost saving for various groups in high-income countries.^40^ A recent study in Cambodia has shown that a simplified model of care could be cost saving compared to no treatment.^41^

When applying country-specific willingness-to-pay thresholds, several studies reported that expanding treatment in the general population would be cost-effective. Our results confirm this assertion that scaling up HCV treatment to the national level as part of an elimination program can be cost-effective.

## 5 ∣ CONCLUSION

This paper provides valuable data on the cost-effectiveness of a national program for scaling up HCV testing, diagnosis and treatment for achieving HCV elimination. Although competition and negotiated pricing have reduced prices, cost continues to limit the reach of DAA therapies in many countries.^42^ Insurers, government and pharmaceutical companies should work together to bring medication prices to the point where all persons in need of treatment are able to afford and readily access these drugs. Local manufacture of generic medicines has the potential to reduce prices further.^3,43^ Alternative funding models, such as using catalytic funding (in which external start-up funds are provided to start a program which will become self-sufficient), have been proposed to improve access to HCV treatment.^10^

Overall, Georgia's HCV elimination program has accomplished an impressive scale-up of treatment, which has already impacted the HCV burden. The analysis shows that the program was highly cost-effective, with the results being useful for decision-making of other countries to help them decide how much they can pay for DAAs or which strategy might be cost effective. Our study findings could contribute to the development of national and global policies as well as contribute to better planning and management of programs around the globe.

## Supplementary Material

Supplement

## Figures and Tables

**TABLE 1:** Characteristics of patients screened for Hepatitis C virus antibodies from 28 April 2015 to 30 November 2017 (770 832 unique persons)

Characteristics	*N*	%
Age		
< 18	84 680	11.0
18–49	421 644	54.7
≥ 50	264 398	34.3
Missing	110	
Sex		
Female	407 874	52.9
Male	362 750	47.1
Missing	208	
Screening site^a^		
Inpatients	277 473	36.3
Outpatients	142 271	18.6
Georgia harm reduction network	4312	0.6
Blood bank	93 346	12.2
Prison	3953	0.5
Antenatal clinic	67 595	8.8
Military recruits	14 538	1.9
Other	161 394	21.1
Missing	5950	
Positive screening test	76 699	10.0

a Site where first screened for those patients screened more than once.

**TABLE 2:** Demographic and baseline clinical characteristics of the patients who initiated HCV treatment from 28 Apr 2015 to 30 Nov 2017 (41 483 unique persons)

Characteristics	*N*	*%*
Groups		
General population	38 825	93.7
Living with HIV (treated at infectious disease hospital)	688	1.6
Incarcerated (Treated at Ministry of correction)	1970	4.7
Age		
< 18	7	0.0
18–49	26 464	63.8
≥ 50	15 012	36.2
Sex		
Female	7358	17.7
Male	34 125	82.3
First treatment regimen		
12 weeks sofosbuvir/ledipasvir	14 867	35.9
12 weeks sofosbuvir/ledipasvir + ribavirin	17 547	42.3
12 weeks sofosbuvir/ledipasvir + ribavirin + interferon	52	0.1
12 weeks sofosbuvir + ribavirin	507	1.2
12 weeks sofosbuvir + ribavirin + interferon	3481	8.4
20 weeks sofosbuvir + ribavirin	579	1.4
24 weeks sofosbuvir/ledipasvir	251	0.6
24 weeks sofosbuvir/ledipasvir + ribavirin	1383	3.3
24 weeks sofosbuvir/ledipasvir + ribavirin + interferon	29	0.1
24 weeks sofosbuvir + ribavirin	2110	5.1
48 weeks sofosbuvir + ribavirin	665	1.6
Missing	12	
Genotype		
GT1	17 998	43.4
GT2	8514	20.5
GT3	14 218	34.3
Other	753	1.8
Calendar year of treatment		
2015	5938	14.3
2016	21 656	52.2
2017	13 889	33.5
Liver disease assessment method		
Elastography	19 424	46.8
FIB-4 only	22 059	53.2

**TABLE 3:** Unit costs of components of screening and treatment program (USD by year)

	2015	2016	2017	Source for cost estimates
Direct costs (per patient treated)				
Screening test^a^	$5.1	$4.1	$22.6	NCDC
MOH share	15%	12%	25%	
Private health facility share	85%	88%	75%	
HCV RNA test^a^	$54.2	$41.9	$42.6	MOH financial reimbursement records
MOH share	34%	33%	34%	
Patient share	66%	67%	66%	
Pre-treatment evaluation with FIB-4 only^a^	$38	$112	$111	MOH financial reimbursement records
MOH share	65%	31%	34%	
Patient share	35%	69%	66%	
Pre-treatment evaluation with elastography^a^	$92	$136	$141	MOH financial reimbursement records
MOH share	35%	30%	34%	
Patient share	65%	70%	66%	
Treatment monitoring visits and blood tests^a^	$176	$104	$27	MOH financial reimbursement records
MOH share	17%	27%	30%	
Patient share	83%	73%	70%	
Non-DAA drugs (ribavirin/interferon)	$313	$48	$33	List price in Georgia
MOH share	100%	100%	100%	
DAA drugs (list price)	$93 008	$72 129	$84 294	
DAA drugs (generic)	$1029	$1043	$1047	
DAA drugs (minimal)	$47	$59	$60	
Indirect costs (total per year)				
Outreach	$70 462	$48 235	$21 880	MOH and NCDC records
Logistics	$35 983	$190 840	$158 661	
MOH share	100%	100%	100%	
Costs for clinic equipment	$16 942	$16 942	$16 942	MOH records
Clinic share	100%	100%	100%	
Liver disease care (per patient receiving care)				
Mild	$318	$281		MOH records from the Management of Infectious Diseases state program
MOH share	86%	885		
Patient share	14%	12%		
Moderate	$427	$392		
MOH share	91%	86%		
Patient share	9%	14%		
Severe^b^	$675.5	$689.8		
MOH share	88%	89%		
Patient share	12%	11%		

Abbreviations: DAA, direct-acting antivirals; MOH, Ministry of Internally Displaced Persons from the Occupied Territories, Labour, Health and Social Affairs; NCDC, National Center for Disease Control.

a Screening, diagnostic and pre-treatment evaluation costs were calculated per patient treated according to a cascade of care. The breakdown of costs between MOH and patients is weighted by the proportion of treatments for patients designated as socially vulnerable, which varied by year (15.3% in 2015, 9.3% in 2016 and 12.3% in 2017). See Data S1 for additional details on variation in patient out-of-pocket costs.

b Severe liver disease includes cirrhosis, ascites and/or encephalopathy and/or hepato-renal syndrome.

**TABLE 4:** Incremental cost-effectiveness ratio (ICER) table for the base case, comparing treatment in the study period to no treatment, including costs to the MOH and generic DAA costs, with costs and outcomes measured over 25 years and discounted at 3% per year

	Cost (USD) per capita		QALY per capita		ICER
Strategy	Total	Incremental	Total	Incremental	Cost/QALY
No treatment	272	-	1340.8	-	-
Treatment	1586	1314	1340.2	0.57	$2285

## Data Availability

The data that support the findings of this study are available on request from the corresponding author. The data are not publicly available owing to privacy or ethical restrictions.

## Abbreviations:

Anti-HCV: HCV antibody
DAA: Direct-Acting Antiviral
GDP: country's gross domestic product
HCC: Hepatocellular Carcinoma
HCV: Hepatitis C virus
HCVcAg: HCV core antigen testing
ICER: incremental cost-effectiveness ratio
PLHIV: People Living with HIV
PWID: People Who Inject Drugs
QALY: quality-adjusted life year
RNA: ribonucleic acid
SVR: Sustained Virologic Response
