# Supplementary material for: Economic evaluation of the Hepatitis C virus elimination program in the country of Georgia, 2015 to 2017
Source: Liver Int. Author manuscript; Available in PMC 2024 Mar 1. (PMC10227952; doi:10.1111/liv.15431)
Supplement: Supplement [file NIHMS1899405-supplement-Supplement.docx]

Supplementary document

**Economic evaluation of the Hepatitis C Virus Elimination program in Georgia, 2015 to 2017**

Table of Contents

[Cost data 2](#_Toc97131584)

[Screening and treatment 2](#_Toc97131585)

[Pharmaceutical costs 2](#_Toc97131586)

[Indirect costs 3](#_Toc97131587)

[Total cost of the treatment 3](#_Toc97131588)

[Cost of care for HCV-associated liver disease 3](#_Toc97131589)

[Supplementary Tables 4](#_Toc97131590)

[Table 1 – Unit cost inputs to model from each calendar year. Cost of screening, HCV Confirmation, pretreatment evaluation with or without elastography, monitoring, non-DAA drugs, and indirect costs. These costs were multiplied by number of patients receiving them and averaged over all patients initiating treatment. 4](#_Toc97131591)

[Table 2 – Vertical program -cost of care by year and by category (pre-cirrhosis, cirrhosis, decompensated cirrhosis) 4](#_Toc97131592)

[Table 3 – Regimen unit costs per dose 5](#_Toc97131593)

[Table 4 Fixed costs 5](#_Toc97131594)

[Table 5 QALY weights Georgia and Chong study 5](#_Toc97131595)

## Cost data

### Screening and treatment

Screening unit cost information was provided by the National Center for Disease Control and Public Health (NCDC), as calculated for regional pilot projects. Screening test costs included: salaries, purchasing of goods and services, business trips etc. It includes the cost of screening for HCV, HIV, and TB together, and entry of results into the screening database. In government-sponsored screening centers (covering outpatient or inpatient testing), the reimbursement cost per test was 0.53 USD, while elsewhere the reimbursement cost was 0.59 USD.

Costs for pretreatment evaluation, monitoring during treatment, and viral load testing were received from MOH based on financial reimbursement to health care providers participating in the treatment program for the full period from 2015 through November 2017 (Supplementary Table 1). Relevant codes, prices, and number of claimed and reimbursed cases were used to calculate the total cost to the MOH in each calendar year. Information from the elimination program treatment database was also used for the number of patients treated. All diagnostics before treatment initiation were reimbursed only once per patient, whilst diagnostics in the monitoring process might be reimbursed twice, in case of re-enrollment in treatment as a result of relapse, reinfection, treatment discontinuation). Additional administrative costs for clinics starting from June 2016 were covered by MOH and included in the reimbursement.

Pretreatment evaluation included HCV RNA quantification, HCV genotyping, complete blood count, ALT, AST, creatinine, bilirubin, albumin, INR, alkaline phosphatase, G-GT, glucose, HBsAg, anti-HBc total, FIB-4 for liver fibrosis assessment (noninvasive tests used to determine the degree of hepatic fibrosis). Patients with FIB-4 score between the lower and upper cut-off values undergo liver elastography to assess fibrosis stage, and a separate reimbursement code and cost for pretreatment evaluation was used for these patients. A weighted average was used to account for the proportion of patients receiving elastography in the total cost of treatment for each year.

The proportion of cost covered by the MOH differed for two categories of patients, with people registered as socially vulnerable with the national government receiving 70% of costs from MOH directly, and the 30% co-payment covered by local city government. For all others, the MOH covered 30% of costs and different shares of co-payments were covered by city government in different regions. Due to the limitation of not knowing details of proportion of co-payment paid out of pocket by patients, in this analysis we allocate all co-payments to the patient. A weighted average was used to calculate the total cost of treatment attributable to the MOH vs patient co-payment in each year.

### Pharmaceutical costs

Information on the number of patients and prescribed regimens was extracted from the national treatment database (“Elim C”). The list prices of donated direct acting antivirals (DAAs) were based on registration prices in Georgia, with the average price of sofosbuvir $630 USD per tablet and sofosbuvir/ledipasvir $941 USD per tablet, with variation across years (Supplementary Table 3). Generics prices sourced from India, as used in the base case scenario, were gathered from interview with healthcare providers treating patients outside of the HCV elimination program, with generic sofosbuvir/ledipasvir priced at $12 USD per tablet and generic sofosbuvir at $7.20 per tablet in 2017. The minimal DAA cost included as an alternative scenario uses production cost-based prices of $0.33 for sofosbuvir and $0.69 for sofosbuvir/ledipasvir as identified in a recent study^[[1]](#footnote-1)^. Some regimens included ribavirin and interferon in addition to DAAs, these drugs were paid for fully by the MOH at 0.10 USD per tablet for ribavirin and 39.91 USD per ampule for interferon. The cost of drugs included in the model accounts for the proportion of patients receiving different regimens, and changes in price year to year.

### Indirect costs

Additional fixed or indirect costs accounted for included drug logistics, purchase, and delivery services (custom clearance, warehousing, drug transportation and escort to the service centers and marking of drugs) and outreach activities by NCDC and MOH. In addition, we included costs for clinic equipment of approximately 1720 USD per facility to 31 service centers for required equipment to provide services within HCV elimination program for which costs were provided by Ministry (Supplementary Table 4).

### Total cost of the treatment

To calculate total cost per patient for each calendar year, each cost component or code was multiplied by the number of cases: number of patients screened, receiving RNA test, pretreatment diagnostics with or without elastography, treatment monitoring, each drug regimen, SVR testing. Total indirect costs were added and this was then divided by the number of patients initiating treatment during the relevant time period. The same method was used for MOH and patient co-payment estimates.

### Cost of care for HCV-associated liver disease

To fully estimate the cost-effectiveness of the HCV elimination program in Georgia, health care costs that would have occurred without the program were also estimated. The data were derived from the “Management of Infectious Diseases” state program database for all the patients enrolled within the state program, which has been operating since 15 February 2011 (Supplementary Table 2). The treatment of some clinical conditions related to HCV infection were partially covered by state funds, which we estimated based on data provided from the MOH for January 2015-December 2017, including ICD10 codes with a description of diagnoses. In this case, to define Chronic HCV, we used ICD 10 code B18.2 and 3 internal codes for reimbursement including (1) Chronic viral hepatitis with the highly active pathologic process, (2) Chronic viral hepatitis with cirrhosis, (3) Chronic viral hepatitis with cirrhosis, ascites and/or encephalopathy, and/or hepato-renal syndrome, and (4) Acute viral hepatitis C. The maximum amount of 279 USD was reimbursed for the acute viral hepatitis cases, a maximum of 271 USD for chronic hepatitis cases with high clinical activity and a maximum of 452 USD for chronic hepatitis cases with cirrhosis, with different co-payments for each category.

## Supplementary Tables

### Table 1 – Unit cost inputs to model from each calendar year. Cost of screening, HCV Confirmation, pretreatment evaluation with or without elastography, monitoring, non-DAA drugs, and indirect costs. These costs were multiplied by number of patients receiving them and averaged over all patients initiating treatment.

| **Year** | **Screening** | | **HCV RNA Confirmatory testing** | | **Pretreatment evaluation** | | **Pretreatment evaluation +Elastography** | | **Monitoring** | | **Non-DAA drugs** | **SVR** | | **Indirect costs** | |
| --- | --- | --- | --- | --- | --- | --- | --- | --- | --- | --- | --- | --- | --- | --- | --- |
|  | SG | SH | MOH | P | MOH | P | MOH | P | MOH | P | MOH | MOH | P | MOH | HCP |
| **2015** | 0.56 | 0.63 | 18.6 | 35.6 | 24.5 | 13.1 | 32.6 | 59.6 | 30.0 | 146.4 | 331.3 | 29.7 | 13.4 | 106445.1 | 50,825 |
| **2016** | 0.52 | 0.57 | 14.0 | 27.9 | 35.0 | 76.5 | 41.4 | 95.5 | 28.5 | 75.6 | 174.5 | 47.8 | 0 | 239074.8 |  |
| **2017** | 0.52 | 0.58 | 14.6 | 28.0 | 37.7 | 73.5 | 48.0 | 93.9 | 8.2 | 19.2 | 288.4 | 48.6 | 0 | 180540.9 |  |

SG- Screening Payed by Government; SH- Screening Bayed by HCP (Other); MOH- Ministry of Health; P-Patient; HCP- Health care provider

### Table 2 – Vertical program -cost of care by year and by category (pre-cirrhosis, cirrhosis, decompensated cirrhosis)

| **Chronic** HCV **ICD 10 B18.2** | | | | | | |
| --- | --- | --- | --- | --- | --- | --- |
| **Total N of cases** | **Amount to be paid by the state program** | **Average by Gov** | **Patient Co-payment** | **Average by Patient per code** | **Reporting year** | **Average Hosp/per year** |
| **Chronic viral hepatitis with highly active pathologic process** | | | | | | |
| **54** | 14734.3 | 272.8 | 2327 | 45.6 | 2015 | 1.02 |
| **49** | 12105.9 | 247 | 1682.2 | 34.3 | 2016 | 1.02 |
| **Chronic viral hepatitis with cirrhosis** | | | | | | |
| **92** | 35680.4 | 387.8 | 3567.7 | 39.2 | 2015 | 1.05 |
| **82** | 27633 | 337 | 4480.8 | 54.6 | 2016 | 1.05 |
| **Chronic viral hepatitis with cirrhosis, ascites and / or encephalopathy, and / or hepato-renal syndrome** | | | | | | |
| **795** | 474827.3 | 597.2 | 62402.5 | 78.3 | 2015 | 1.14 |
| **670** | 409248.9 | 610.8 | 52945 | 79 | 2016 | 1.13 |

### Table 3 – Regimen unit costs per dose in USD

| Drug | Year | | |
| --- | --- | --- | --- |
| **Category** | **2015** | **2016** | **2017** |
| Sof (list price) | 660.60 | 602.60 | 557.80 |
| Sof/LED (list price) | 938.20 | 815.30 | 964.10 |
| Sof (generic) |  |  | 7.20 |
| Sof/Led (generic) |  |  | 12.00 |
| Sof (minimal) |  |  | 0.33 |
| Sof/Led (minimal) |  |  | 0.69 |
| RBV | 0.10 | 0.10 | 0.10 |
| INF | 37.90 | 34.60 | 43.00 |

### Table 4 Fixed costs

| **Fixed costs (USD)** | **Year** | | | |
| --- | --- | --- | --- | --- |
| **Category** | **2015** | **2016** | **2017** | **Total** |
| **Costs for equipment** | NA | NA | NA | 50825.3 |
| **Outreach** | 70461.8 | 48235.1 | 21879.8 | 55563.9 |
| **Logistics** | 35983.3 | 190839.7 | 158661.1 | 152365.2 |

### Table 5 QALY weights Georgia and Chong study^[[2]](#endnote-1)^

| **Chong QALY** | **Chong mean** | **Chong low** | **Chong High** | **Georgia mean** | **Georgia Low** | **Georgia high** |
| --- | --- | --- | --- | --- | --- | --- |
| **Mild** | 0.76 | 0.68 | 0.83 | 0.83 | 0.81 | 0.85 |
| **Moderate** | 0.76 | 0.68 | 0.83 | 0.81 | 0.77 | 0.85 |
| **Cirrhosis** | 0.74 | 0.66 | 0.83 | 0.74 | 0.68 | 0.8 |
| **Decompensated Cirrhosis** | 0.66 | 0.46 | 0.86 | N/A | N/A | N/A |
| **HCC** | 0.65 | 0.44 | 0.86 | N/A | N/A | N/A |
| **PWID^[[3]](#endnote-2)^** | 0.79 | N/A | N/A | N/A | N/A | N/A |
| **SVR** | 0.83 | 0.77 | 0.9 | N/A | N/A | N/A |

1. <https://www.sciencedirect.com/science/article/pii/S2055664020300017#tbl1> Barber et al 2020 [↑](#footnote-ref-1)
2. <https://www.ncbi.nlm.nih.gov/pubmed/12650799> [↑](#endnote-ref-1)
3. Martin et al., “Cost-Effectiveness of HCV Case-Finding for People Who Inject Drugs via Dried Blood Spot Testing in Specialist Addiction Services and Prisons.” [↑](#endnote-ref-2)
